# Mechanical evaluation of mandibular fractures stabilized with absorbable implants or intraoral splints in cats

**DOI:** 10.3389/fvets.2024.1525586

**Published:** 2025-01-07

**Authors:** Ana C. Castejon-Gonzalez, Chet S. Friday, Michael W. Hast, Alexander M. Reiter

**Affiliations:** ^1^Department of Clinical Sciences and Advanced Medicine, School of Veterinary Medicine, University of Pennsylvania, Philadelphia, PA, United States; ^2^Department of Orthopaedic Surgery, University of Pennsylvania, Philadelphia, PA, United States; ^3^Department of Mechanical Engineering, University of Delaware, Newark, DE, United States

**Keywords:** feline, mandibular fracture, absorbable plate, absorbable mesh, mechanical testing, deflection angle

## Abstract

**Introduction:**

The goal of this cadaver study in cats was to compare the mechanical properties of intact mandibles (C) with mandibles whose simulated fracture was located between the third and fourth premolar teeth and repaired with four possible treatments: (1) Stout multiple loop interdental wiring plus bis-acryl composite intraoral splint (S); (2) modified Risdon interdental wiring plus bis-acryl composite intraoral splint (R); (3) ultrasound-aided absorbable fixation plate (P); and (4) ultrasound-aided absorbable fixation mesh (M).

**Materials and methods:**

Thirty feline mandibles were randomly assigned to the control and treatment groups. Mandibles were loaded by cantilever bending on the canine tooth, first in non-destructive cyclic loading followed by destructive ramp-to-failure loading.

**Results:**

Cyclic loading showed no differences between the treatment groups in angular deflection (a measure of sample flexion under non-destructive loads); however, the R group had significantly higher angular deflection than the C group. In destructive testing, no differences in mechanical properties were found between the treatment groups; however, all treatment groups demonstrated significantly lower maximum bending moment, bending stiffness, energy to failure, and maximum force when compared to the control group. The main mode of failure of the intraoral splint groups (S and R) was fracture of the bis-acryl composite (50%), and the main mode of failure of the absorbable fixation groups (P and M) was fracture of the pins (91.7%).

**Discussion:**

Intraoral splint and absorbable fixation methods have low strength and stiffness. The four treatments tested provided similar stabilization of mandibular fractures located between the third and fourth premolar teeth.

## 1 Introduction

Mandibular fractures occur frequently in cats with maxillofacial trauma (72–86.7% of cases), with approximately one-third involving the canine, premolar, and molar regions (1, 2). Despite the high prevalence of multiple fractures, most mandibular fractures of the tooth-bearing areas are unilateral (2). Mandibular fractures are one of the most common injuries to undergo surgery in trauma patients (3). Repair is indicated to reduce pain, stabilize the fracture fragments, promote bone healing, recover masticatory function, and avoid malocclusion (4). General interventions for mandibular fracture repair include non-invasive or minimally invasive treatments [muzzling, intraoral splint, lingual arch bar, elastic training, labial sutures through buttons, maxillomandibular fixation, and bi-gnathic encircling and retaining device (BEARD)] or invasive treatments (miniplates, intraosseous wires, and external fixators). The selected treatment depends on the location and type of fracture, the availability of needed equipment, instruments, and materials, and the skills of the oral surgeon (1, 4–8).

Among veterinary dentists and oral surgeons, intraoral wire-reinforced bis-acryl composite splints remain the treatment of choice to stabilize fractures of the mandibular body if teeth are present rostral and caudal to the fracture line (4). Excellent results have been reported in the only retrospective study of mandibular fractures treated with intraoral splinting in cats (9). In that study, only three cases were in the body of the mandible (9). While there are multiple interdental wiring techniques described (Stout multiple loop, Risdon, Essig, Ivy loop, and crossover) (10, 11), to the best of our knowledge, there are no studies evaluating the effectiveness of the different configurations in cats. A cadaveric study in dogs showed that the mechanical properties of the crossover and Stout multiple loop configurations were comparable (10). Furthermore, bone healing in mandibular fractures repaired with different types of intraoral splints (crossover, Stout, and Risdon) in small breed dogs was achieved at similar time periods (2.3 ± 0.7 months) (12).

The use of titanium miniplates has been reported in experimental studies and case reports for fractures distal to the mandibular fourth premolar tooth or distal to the first mandibular molar tooth (6, 13–15). The ideal location for plate placement in the mandible of cats is the ventral third, as the area avoids damage to the teeth and provides sufficient bone thickness for the screws (14). However, the screws may damage the mandibular canal and its neurovascular contents (14), making the use of miniplates a less preferred technique (4). Furthermore, although not frequently reported, miniplates may need to be removed secondary to plate exposure, infection, or loosening of the implants (16, 17).

Recently, absorbable implants (Resorb X^®^, KLS Martin, Germany) and pins (SonicPins^®^, KLS Martin, Germany) based on poly-(50% D, 50% L)-lactic acid (PDLLA) have been introduced to the veterinary market for maxillofacial applications. They are recommended for fractures of the mid and upper face or bone augmentation in areas that do not support any load or masticatory forces. The same plates and pins have been successfully used in mandibular fractures in children (18–20). The implants (plates or meshes) are fixed to the bone by pins instead of screws. The pins are inserted into the pilot holes by ultrasonic activation. The micro-vibrations melt the outer surface of the pins, which quickly solidify after discontinuing the activation, welding the pins to the porous structure of the cortical and cancellous bone. The heads of the pins are also welded to the plate or mesh (21). The implants are safe and biocompatible and do not cause foreign body reactions or inflammation beyond the reaction expected during healing (22). They maintain the initial strength for 8–10 weeks and are completely degraded by hydrolysis in approximately 6–12 months. The degradation occurs at the same speed as the ossification, increasing progressively the load in the bone and avoiding stress shielding (23, 24). Other reported advantages of absorbable implants include their faster and easier application compared to titanium plates and screws and lack of interference with radiology because they are radiolucent. They are also ideal for thin cortical and cancellous bone because they weld to bone (25). The main disadvantage is that they are weaker and less stiff than titanium plates; therefore, they may not be sufficient to support the forces of mastication and provide enough stabilization to obtain bone healing.

Neither intraoral wire-reinforced bis-acryl composite splints nor absorbable implants have been studied previously in cats. Therefore, the goals of this study were (1) to evaluate the mechanical properties of four different configurations used to repair mandibular fractures created by a transverse osteotomy between the third and fourth premolar teeth and (2) to compare the application time between constructs and the possible injury of important anatomical structures between two types of absorbable implants.

## 2 Materials and methods

Cadaver heads with fully erupted teeth of cats euthanized for reasons not related to this study were obtained. Cadaver heads with incompletely erupted permanent teeth, periodontal disease, and missing teeth were excluded. Age, weight, and gender were not available. Thirty mandibles were obtained from 17 cadaver heads. Mandibles were randomly assigned to one of the following five groups: (1) Control (C): intact mandibles; (2) Stout (S): intraoral splint with Stout multiple loop wire configuration and bis-acryl composite; (3) Risdon (R): intraoral splint with modified Risdon wire configuration and bis-acryl composite; (4) Plate (P): absorbable plate and four pins; and (5) Mesh (M): absorbable mesh and six pins. Mandibles assigned to S, R, P, and M groups were cut between the third and fourth premolar teeth with a diamond disk (diamond-coated disk HP, 0.17 mm, Miltex, York, PA) after making an intraoral incision. The cut simulated a transverse fracture. The mandibles were excised and detached from all soft tissue attachments after the repair of both mandibles was completed. Radiographs were obtained after harvesting. The specimens were wrapped individually in a moist gauze and frozen until biomechanical testing.

### 2.1 Clinical procedure (all procedures were performed by the same investigator, AC)

The intraoral incision performed to approach the mandible for osteotomy was sutured closed prior to stabilization.

#### 2.1.1 Intraoral wire-reinforced bis-acryl composite splints (groups S and R)

The teeth included in the splint were scaled and polished with coarse pumice.

**Figure 1:**
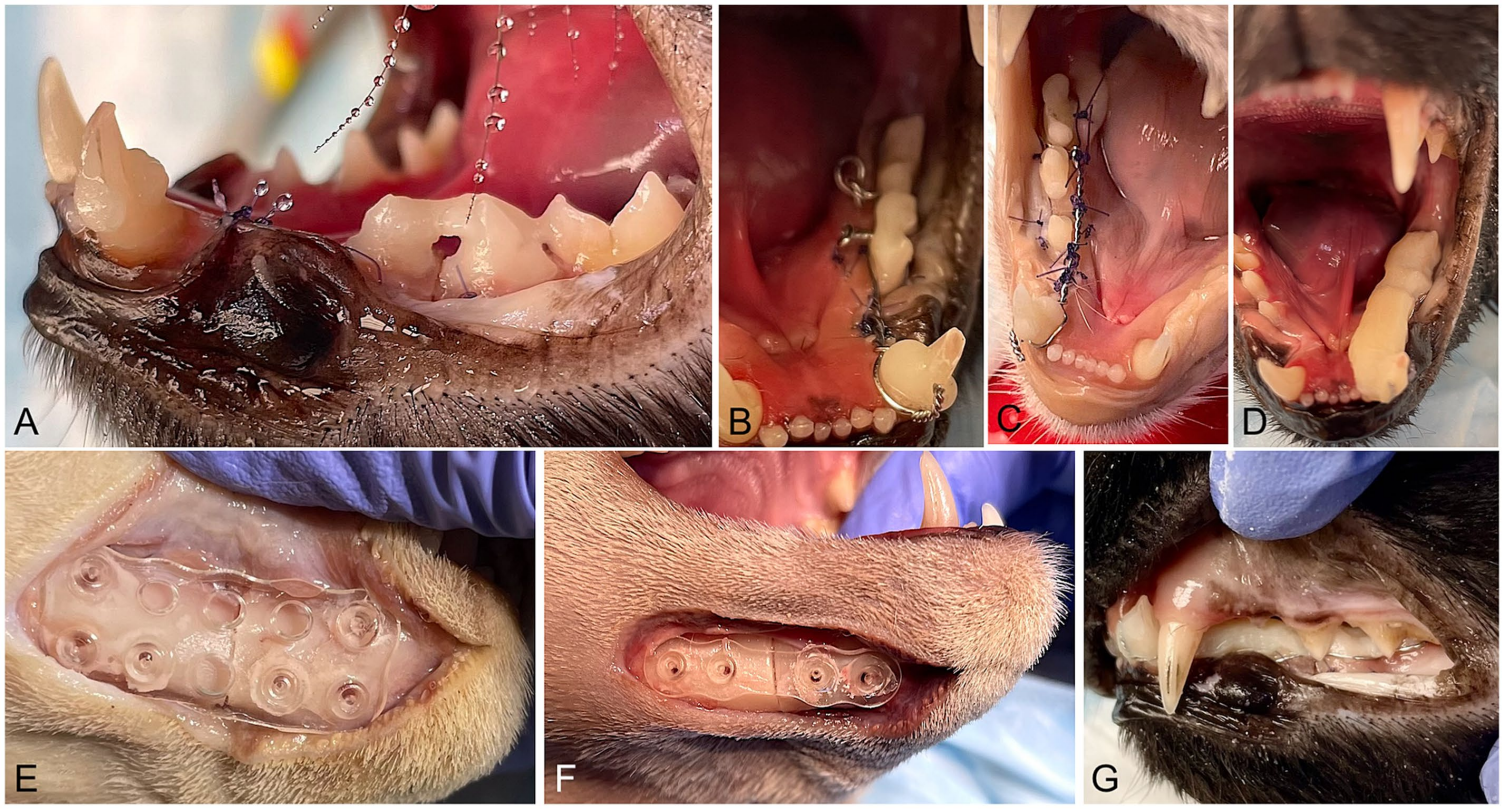
Intraoperative images of the procedures. **(A)** Initial steps of an intraoral splint with a Stout multiple loop configuration. Bridges made of dental composite between the third and fourth premolar and fourth premolar and molar teeth are visible. The mandibular canine tooth has composite buttons in the mesiolabial and distolingual aspects. **(B)** A 26G wire was placed in a Stout multiple-loop configuration. The wire was passed under the bridges and buttons, and loops were placed lingual to the teeth. **(C)** A 26G orthopedic wire was looped around the first molar and canine teeth. The standard Risdon configuration was modified by placing polydioxanone sutures through the gingiva and around the wire. **(D)** Stout and Risdon groups: bis-acryl composite was applied on the teeth and the wire, creating the final shape of the intraoral splint. The intraoral splint has approximately the same width and height in all its length. **(E)** Half 26 × 26 mm absorbable mesh attached to the lateral aspect of the mandible with six pins (two ventral and one dorsal on each side of the mandibular fracture). **(F)** 1 mm thick absorbable 4-hole plate attached with four absorbable pins to the ventral and lateral surface of the mandible **(G)**. Occlusion after fracture repair with intraoral splints.

- Stout group (S): Buttons made from resin-based dental composite (Omnichroma Flow^®^ Tokuyama, Tokyo, Japan) were placed on the crowns in the distal aspect of the first molar tooth, mesial aspect of the third premolar tooth, and labial and lingual aspects of the canine tooth, and bridges made of the same dental composite were placed between the premolar and molar teeth. All composite buttons and bridges were applied after etching the tooth surface for 20 s (40% orthophosphoric acid, Patterson Veterinary), rinsing and drying the tooth surfaces, and applying light cured unfilled resin (Prime and Bond^®^NT, Dentsply Sirona, Milford, DE, USA). Then, a 26G orthopedic wire (Surgical steel DS26, Ethicon, Somerville, NJ, USA) was placed between the canine and first molar teeth using a Stout multiple-loop configuration. The wire was passed under the composite buttons and bridges with the loops tightened lingual to the teeth. The teeth were etched again for 20 s, the surfaces of the teeth were rinsed and air-dried for 5 s, and a self-curing bis-acrylic composite (Protemp™Plus, 3 M, Germany) was applied over the teeth and wire with a homogenous thickness and height along the whole splint area (from canine to first molar teeth). The splint was reduced and shaped with an acrylic bur (Goldies Carbide Bur 47XC, Dedeco International Inc., Long Eddy, NY, USA) to obtain complete closure of the mouth (Figure 1).
- Risdon group (R): The modified Risdon technique used in this study will be referred to as Risdon throughout the manuscript. Composite buttons distal to the mandibular molar and canine teeth and a bridge between the fourth premolar and first molar teeth were applied as in the Stout group. A 26G orthopedic wire was looped around the mandibular first molar tooth and twisted lingually to reach the canine tooth. 4/0 polydioxanone (PDS II, Ethicon, Somerville, NJ, USA) was used to anchor the wire to the gingiva in interdental spaces and the diastema between the canine and third premolar teeth (26). When it was not possible to attach the wire to the gingiva (i.e., at the fracture site), additional loops of PDS around the wire were used to create a scaffold to support the bis-acrylic composite. The self-curing bis-acrylic composite was placed over the wire and the teeth and shaped as in the Stout group (Figure 1).

#### 2.1.2 Absorbable implants (groups P and M)

A ventral approach was used to place the implants. After exposure of the mandible, the plate or mesh (Resorb X^®^, KLS Martin, Germany) was adapted to the surface of the mandible after a warm water bath (Xcelsior^®^ water bath) following the manufacturer’s recommendations. The implant was positioned on the reduced fracture, and pilot holes for the pins were made using a battery mini-driver and the corresponding drill bit. The pins (SonicPins^®^, KLS Martin, Germany) were inserted by activating the sonotrode (BoneWelder^®^ Vet System, KLS Martin) and applying light axial pressure until the head of the pin melted with the implant. The surgical approach was closed in two layers (muscular and intradermal) using absorbable suture material.

- Plate group (P): A 4-hole PDLLA plate was fixed on the lateral surface of the mandible with 5-mm long × 1.6 mm in diameter PLA pins (2 on each side of the fracture) as ventral as possible to avoid damage to the roots of the premolar and canine teeth (Figure 1).
- Mesh group (M): A half 26 × 26 mm PDLLA mesh plate was fixed on the lateral aspect of the mandible with six 5 mm-long × 1.6 mm in diameter pins (2 pins ventral and 1 pin dorsal in each fragment) (Figure 1).

The timer for the intraoral wire-reinforced bis-acryl composite splints was set to start just before scaling the teeth and stopped after reducing/shaping the splint and confirming that the mouth could be closed completely. For the absorbable implants, the timer was set just before starting the ventral incision and stopped after suturing the skin. After each repair, occlusion was confirmed to ensure the mouth could be closed completely.

Lateral radiographs (size 4 phosphor plates, SCAN X, AllPro Radiology) of the mandibles following harvesting were obtained to measure the length (distance from the first incisor tooth to the ventral aspect of the condylar process) and the height (distance from the alveolar margin to the ventral border mesial to the fourth premolar tooth) of the mandible. The radiographs were used to evaluate the location of the pins (whether they overlapped with the mandibular canal, teeth, or periodontal ligament) based on radiolucent areas corresponding to the pilot holes in the bone.

### 2.2 Mechanical testing

Mechanical testing was performed on an electromagnetic test frame (TA Instruments ElectroForce 3,550; Eden Prairie, MN, USA) using a 15kN load cell. The samples were loaded in a cantilever bending at the canine tooth to replicate the forces applied to the mandible during biting/mastication, consistent with established protocols (14, 15). In preparation for mechanical testing, the samples were thawed at room temperature 1% phosphate-buffered saline (PBS). The caudal end of each mandible was potted in Field’s metal, ensuring that the canine tooth extended 30 mm from the potted area, thus providing a consistent moment arm length across all samples. The potted samples were then attached to the test frame using custom fixtures and aligned such that the load would be applied directly to the canine tooth. Loading was performed using a custom fixture with a cavity that matched the shape of the tooth, ensuring uniform loading to avoid cracking of the tooth before the failure of the construct. During testing, 3D interfragmentary motions were recorded with a 6-camera 3D tracking system (OptiTrack Motion Track Systems, NaturalPoint Inc., Corvallis, OR, USA). Single retroreflective 5 mm diameter markers were adhered to both sides of the fractured bone using an adhesive. The 6-camera motion tracking system (Optitrack info) tracked the relative motion of the two markers with a resolution of 0.2 mm after calibration. At rest, a virtual axis was established between the two markers, and the angle relative to horizontal was established, serving as the global reference frame. When vertical loads were applied to the canine tooth, the same approach was used to calculate the angle of the two markers relative to horizontal. The calculated difference between those two angles represented the angular displacement between the rostral and caudal bony fragments.

The mechanical testing protocol consisted of a non-destructive cyclic phase and a destructive ramp-to-failure phase. The cyclic phase loaded the sample between non-destructive forces (1–25 N) for 10 cycles at 1 Hz, allowing for the quantification of rotational deformation of the construct during cyclic loading and mechanical properties during failure. Non-destructive forces (1–25 N) were selected as 25–30% of the maximum estimated bite force in cats (27). Calculations were made from outputs from the test frame and optical tracking data. Specifically, interfragmentary motion was defined as the change in angle between tracking points during cyclic loading. Higher angular deflections represented more flexion in the sample when loaded to non-destructive forces.

**Figure 2:**
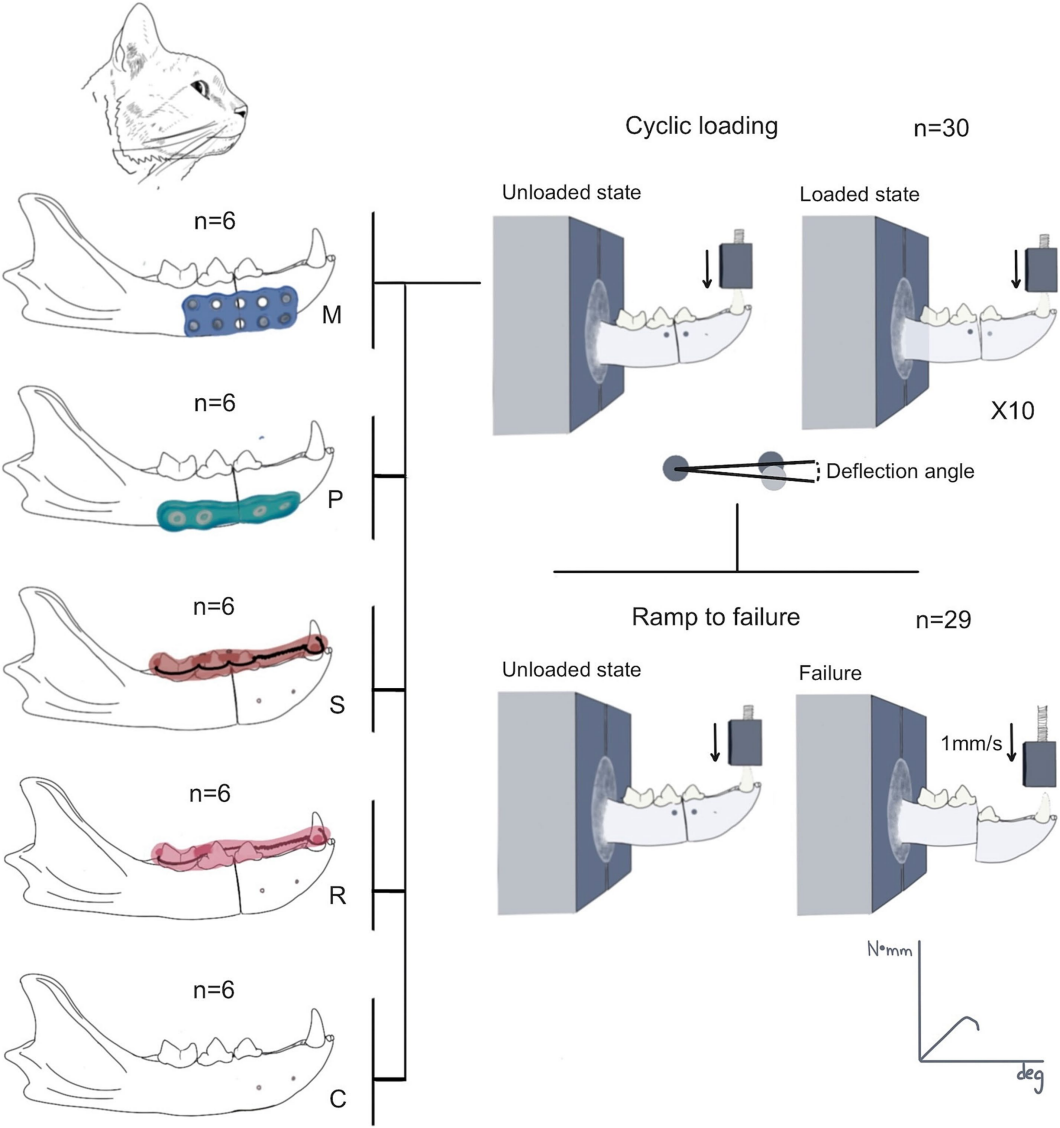
Summary of the research protocol. Thirty mandibles were assigned to one of the groups (control = C; Stout multiple loop = S; modified Risdon = R; plate = P; and mesh = M). The mandibles were harvested after the procedure for mechanical testing. All mandibles of each group were tested during the cyclic loading testing. The deflection angle during the cyclic loading was quantified in 10 cycles, followed by the ramp-to-failure mechanical testing. One tracking point (circle) was placed in each fragment. The angle resultant from the vertical displacement of the rostral tracking point between the loaded and unloaded state was used to quantify the deflection angle. One mandible failed during cyclic loading and was not tested in the ramp-to-failure test.

**Figure 3:**
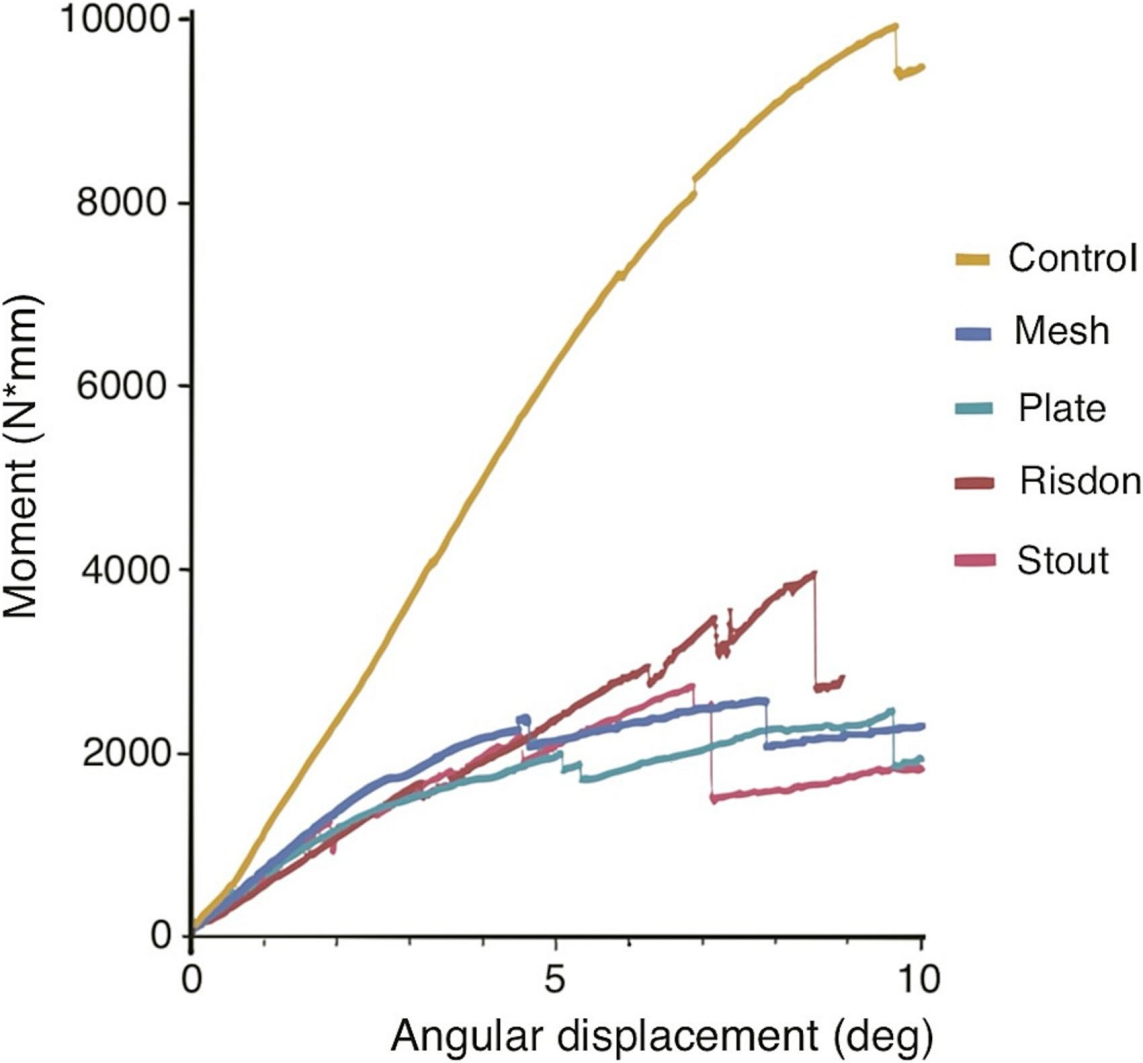
Bending moment-angular displacement curve (averaged by group). Maximum bending moment, bending stiffness (slope of the curve), and energy to failure (area under the curve) of the intact mandibles (control group) were significantly different from the ones of the four treatment constructs (Stout, Risdon, Plate, and Mesh) (see Figures 5, 6).

The destructive phase involved loading the sample to failure at a rate of 1 mm/s to evaluate the structural and failure properties of the construct. Failure was defined as the fracture of the mandible or the repair construct, resulting in a 50% decrease in force. Parameters calculated included maximum force (N), maximum bending moment (N*mm), maximum angular displacement (deg), bending stiffness (N*mm/deg., slope of the moment-angular displacement curve), and energy to failure (N*mm*deg., area under the moment-angular displacement curve) (Figures 2, 3). All data processing was performed using custom MATLAB scripts (MathWorks, Natick, MA, USA).

Mandibles were inspected grossly by two evaluators (AC and CF) to identify the mode of failure of the splints (adhesive, cohesive, or adhesive and cohesive) and the absorbable implants (fracture of the implant and fracture of the pins).

### 2.3 Statistical analysis

The length and height of the mandibles were measured to identify variability between the mandibles of each group and analyzed using the one-way ANOVA test. The normality of the data was assessed using the Shapiro–Wilk test. If the data followed a normal distribution, the groups were compared using the one-way ANOVA with Tukey’s *post-hoc* analysis. When one group or more did not have a normal distribution, a non-parametric test (Kruskal–Wallis test) was used to analyze the difference between the group means. Student’s *t*-test was used to evaluate differences between the number of pins in the mandibular canal, periodontal ligament, or tooth roots between the mesh and plate groups. Bending stiffness and maximum bending moment were compared to the number of pins in the mandibular canal using Pearson’s correlation. *p* < 0.05 were considered statistically significant. Statistical analysis was performed with GraphPad Prism 10.0.

## 3 Results

There were no significant differences in the length and height of the mandible between groups (C, P, M, R, and S).

### 3.1 Experimental surgical procedure

The procedure was longer in both groups of intraoral splinting (S and R) compared to the absorbable implants (P and M). When compared to the duration of the surgical procedure of each group to the plate group (P), Stout (S) application was 2x longer, Risdon (R) was 1.7x longer, and mesh (M) was 1.5x longer. Significant differences were found between M and P, M and S, P and R, and P and S (Table 1).

**Table 1:** Duration of the surgical procedure.

Group	Procedure time mean ± SD (min)	ANOVA test for comparison between groups	
		*Groups*	*P*-value
Mesh	35.4 ± 8.4	Mesh vs. Plate	0.0116*
Plate	23.4 ± 3.0	Mesh vs. Risdon	0.5631*
Risdon	39.95 ± 5.0	Mesh vs. Stout	0.0468*
Stout	45.2 ± 6.1	Plate vs. Risdon	0.0006*
		Plate vs. Stout	<0.0001*
		Risdon vs. Stout	0.4480

*The difference was statistically significant (*p* < 0.05).

**Figure 4:**
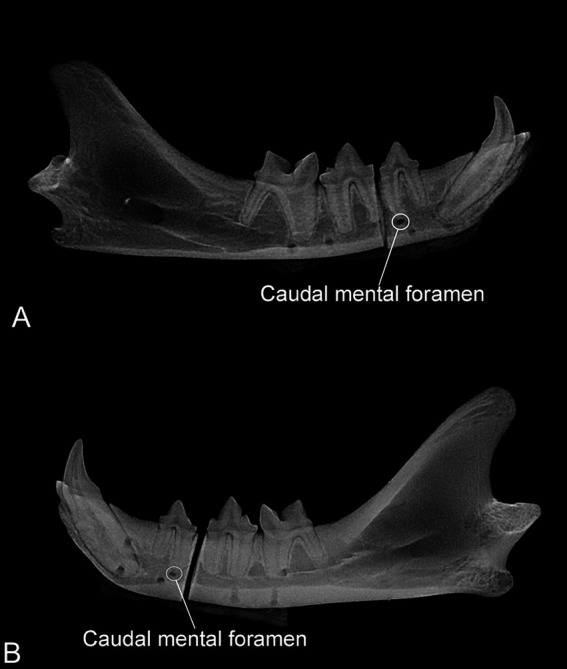
**(A)** Radiograph of one mandible assigned to the plate group. Four radiolucent circular areas are visible in the ventral aspect of the mandible partially overlapping with the mandibular canal. Note that the caudal mental foramen is not aligned with the other four radiolucent areas. **(B)** Radiograph of one mandible assigned to the mesh group. Six circular radiolucent areas are visible in each fragment. One caudal and dorsal and two rostral (one dorsal and one ventral) radiolucent areas are overlapped with the first molar and canine teeth, respectively.

The mean number of pins in the mandibular canal was 2.6 ± 1.0 and 1.8 ± 1.2 for the P and M groups, respectively, but this difference was not significant (*p* = 0.22). There was a significant difference between the number of pins overlapped with the tooth roots in the P (0.3 ± 0.5) and M groups (1.5 ± 1, *p* = 0.0346). The mean number of pins overlapped with the periodontal ligament was 0.8 ± 0.9 and 0.5 ± 0.5 in the P and M groups, respectively, but the difference was not significant (*p* = 0.48) (Figure 4).

### 3.2 Mechanical results

The mean and SD of the length (60.41 ± 4.2 mm), height (9.68 ± 0.74 mm), and moment arm (32.12 ± 2.56 mm) of the mandibles were consistent with no significant differences between groups. One of the mandibles in the plate group (P) failed during the cycling loading and was removed from the mechanical analysis (analysis of the ramp-to-failure testing and cyclic loading testing). One mandible in the Stout group (S) and another in the Mesh group (M) were excluded from the cyclic loading testing analysis because there was excessive noise from the motion-tracker points.

**Figure 5:**
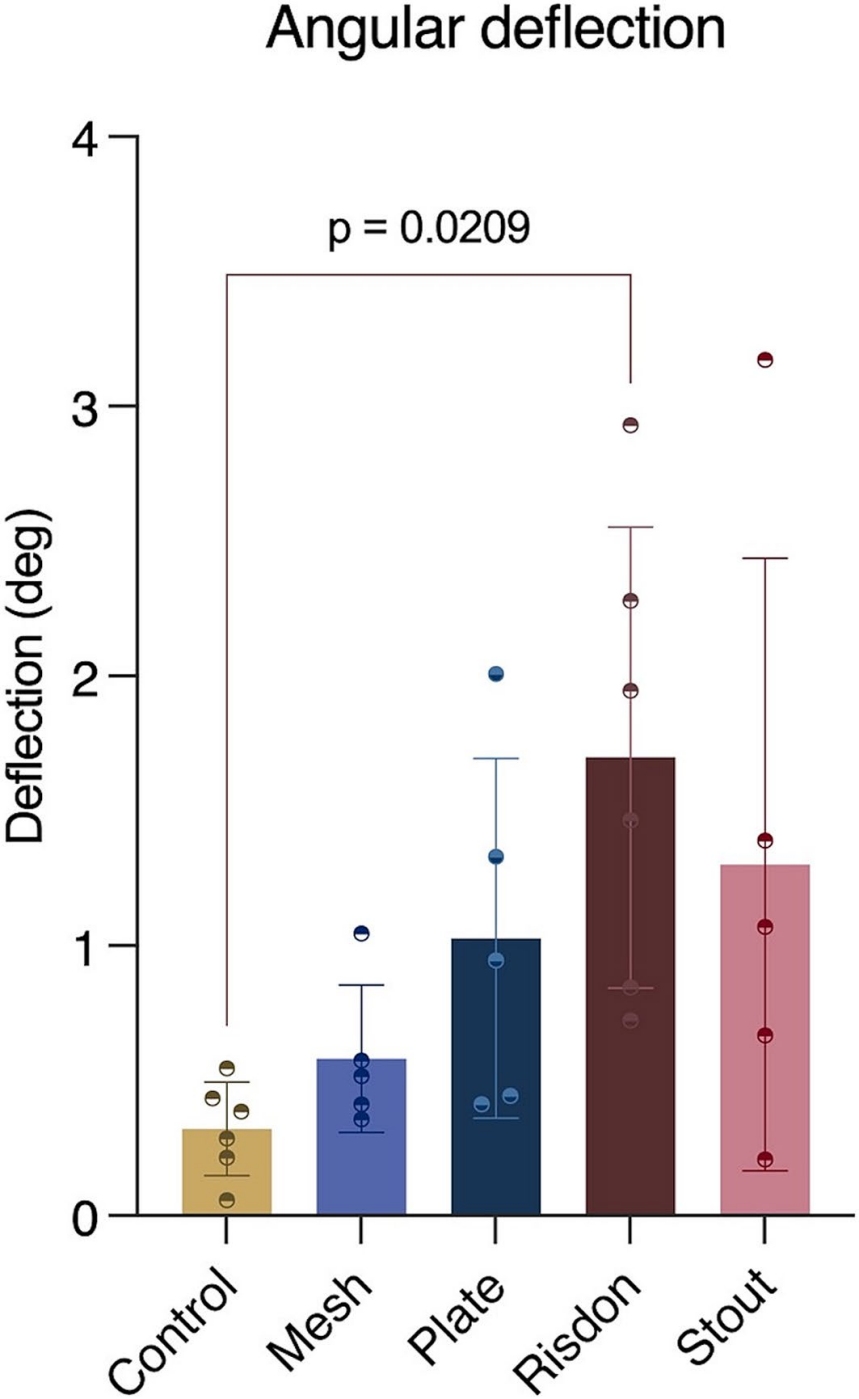
Mechanical testing (cyclic loading). Comparison of the angular deflection (deg) between the experimental groups (bar length = mean; error bar (SD); dots = individual values). Angular deflection of the Risdon configuration was greater than any of the groups but only significantly different from the control group.

Analysis of angular deflection during non-destructive cyclic loading showed no differences between any of the treatment groups (S, R, P, and M). However, the Risdon group (R) had higher angular deflection (1.69 ± 0.85 deg) compared to the control group (C) (0.32 ± 0.17 deg) (Table 2) (Figure 5).

**Table 2:** Mechanical parameters (mean and SD).

Variable	Control	Mesh	Plate	Risdon	Stout	*p*-value
Maximum force (N)	292.4 ± 106.8*	84.87 ± 19.79*	78.31 ± 7.031*	99.88 ± 40.36*	91.04 ± 34.07*	<0.0001
Energy to failure (N*mm*deg)	65.91 ± 23.78*	14.22 ± 8.63*	13.23 ± 6.21*	10.29 ± 6.27*	11.62 ± 5.89*	<0.0001
Maximum bending moment (N*mm)	9,843 ± 3548*	2,682 ± 656.8*	2,604 ± 345.3*	3,076 ± 1248*	2,860 ± 1168*	0.0002
Maximum displacement (mm)	7.05 ± 3.55	4.20 ± 1.97	4.82 ± 1.96	3.34 ± 1.43	3.87 ± 1.48	0.07
Maximum angular displacement (deg)	12.26 ± 6.59	7.9 ± 4.24	8.38 ± 3.57	6.14 ± 2.42	7.36 ± 3.41	0.24
Bending stiffness (N*mm/deg)	1,385 ± 402.5*	661 ± 279.2*	636.7 ± 166.7*	567.7 ± 127.3*	639 ± 35*	0.0002
Deflection angle (deg)	0.32 ± 0.17^	0.58 ± 0.27	1.02 ± 0.66	1.69 ± 0.85^	1.30 ± 1.13	0.02
Moment arm (mm)	33.67 ± 1.61.	31.65 ± 2.84	33.20 ± 2.30	30.95 ± 1.98	31.30 ± 3.32	0.27

*Significant difference (one-way ANOVA, *p* < 0.05) between the control group (intact mandibles) and four treatment groups (Stout, Risdon, Plate, and Mesh). ^Significant difference (one-way ANOVA, *p* < 0.05) between control and Risdon groups.

**Figure 6:**
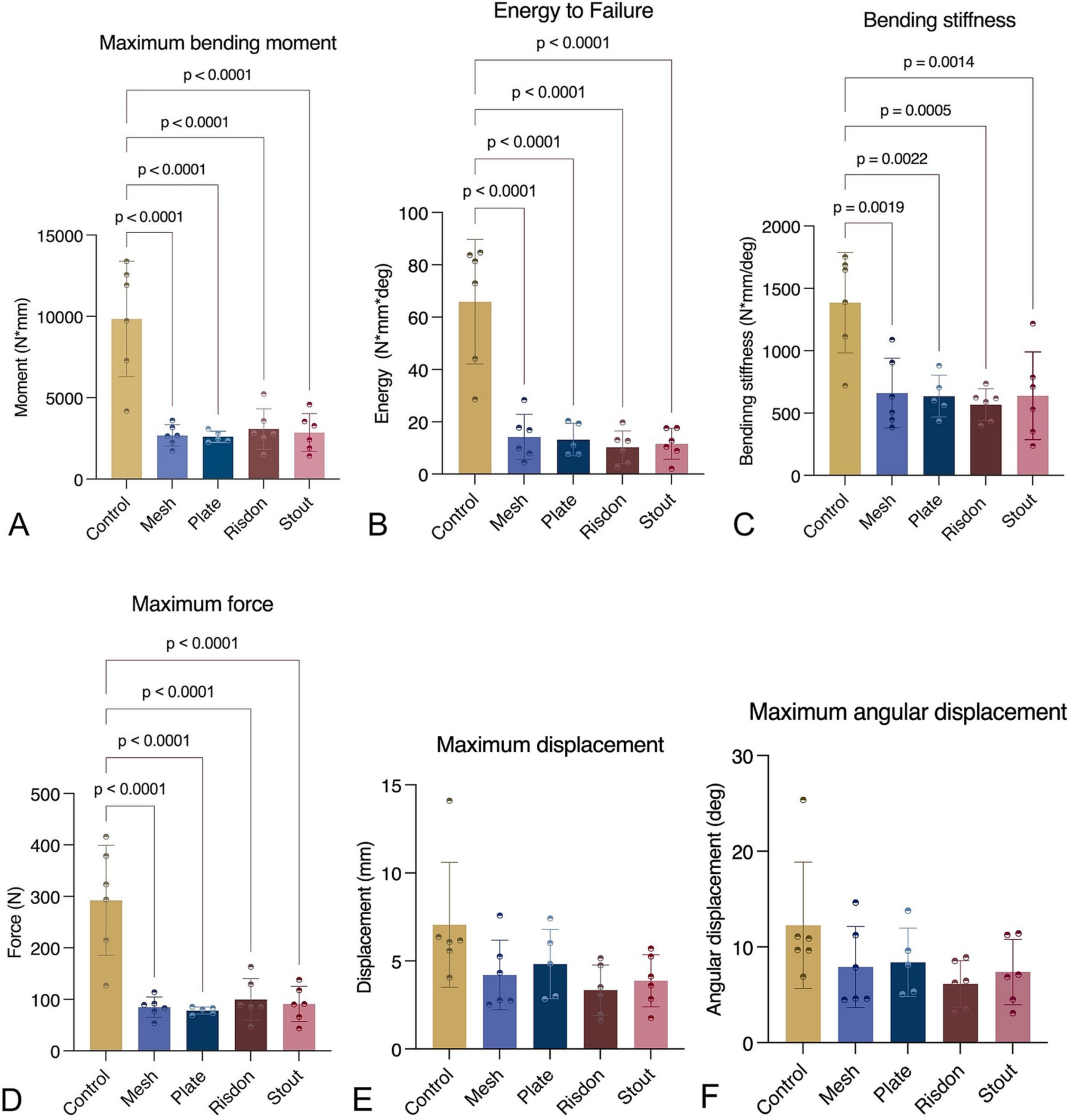
Mechanical testing (ramp to failure). **(A)** Maximum bending moment. **(B)** Bending energy to failure. **(C)** Bending stiffness. **(D)** Maximum force. **(E)** Maximum displacement. **(F)** Maximum angular displacement. Intact mandibles (control) were stronger and stiffer than any of the other groups (*p* < 0.05). All parameters, except maximum displacement and maximum angular displacement, were normally distributed and assessed using ANOVA with Tukey’s *post-hoc* analysis. Maximum displacement and maximum angular displacement were analyzed using the Kruskal–Wallis test.

Destructive testing demonstrated that maximum bending moment, bending energy to failure, bending stiffness, and maximum force were significantly higher in intact mandibles (C) as compared to all four treatment constructs (S, R, P, and M). However, there were no significant differences between any of the intraoral splints and absorbable implants. Maximum displacement and angular displacement were not significantly different between any of the five groups (Kruskal–Wallis non-parametric test) (Table 2 and Figure 6).

Correlations between the number of pins invading the mandibular canal and the bending stiffness (*p* = 0.77) or the bending moment (*p* = 0.47) were not found.

### 3.3 Mode of failure

**Figure 7:**
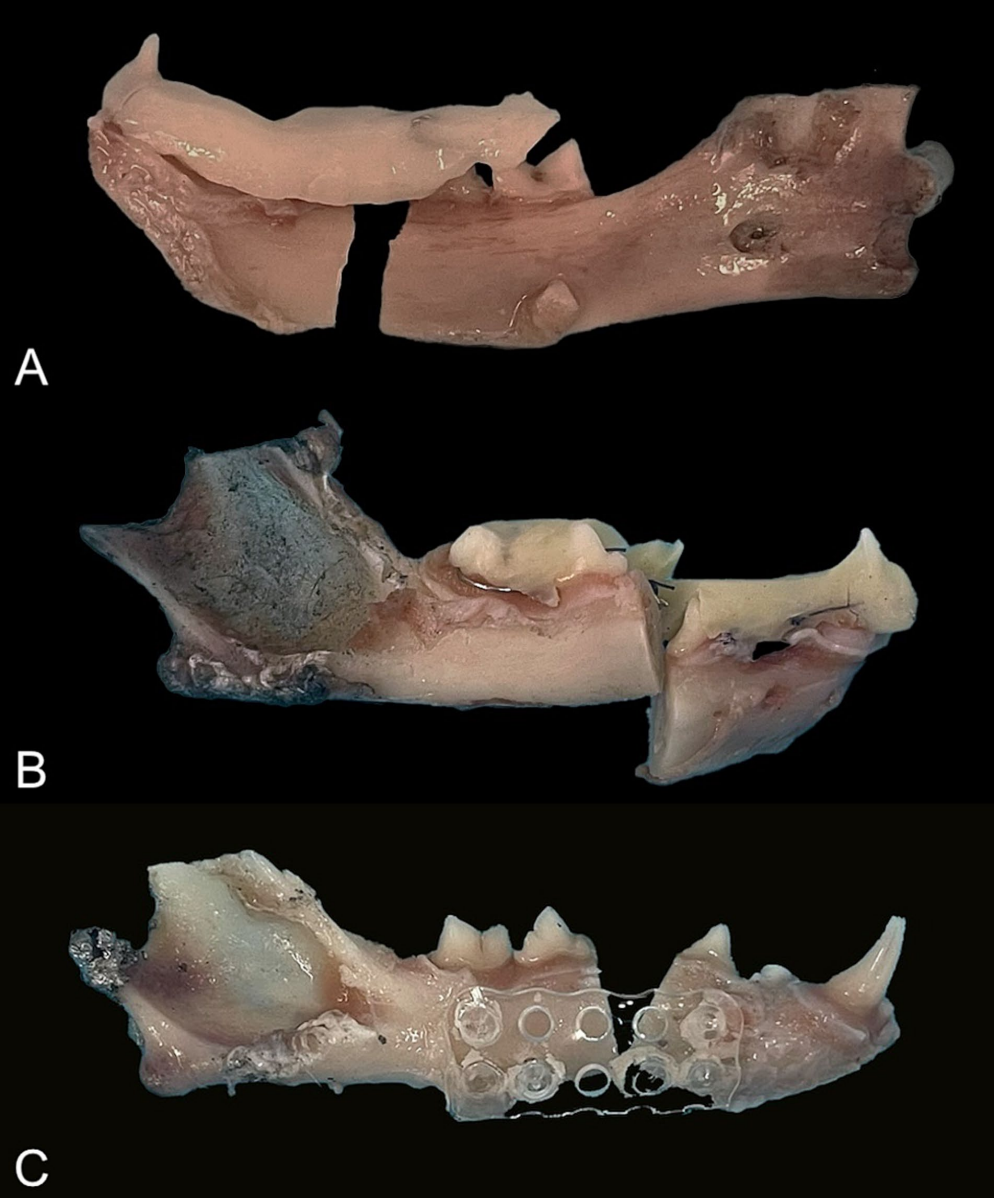
Mode of failure. **(A)** Splint: adhesive failure. **(B)** Splint: cohesive failure. **(C)** Mesh: pins fractured in the rostral fragment.

The intraoral splints failed due to separation from the teeth (adhesive failure, *n* = 3), fracture of the bis-acrylic composite adhesive (cohesive failure, *n* = 6), or both (*n* = 3). Fractures of the splints occurred frequently at the fracture site (between the third and fourth premolar teeth), adhesive failure occurred at the first molar tooth, and combined adhesive and cohesive failure occurred at the fracture site and teeth adjacent to it (Figure 7). The wire did not break in any of the constructs.

The main mode of failure of the plate (P; *n* = 5) and mesh (M; *n* = 4) constructs was the fracture of all the pins at the rostral or caudal bone fragment. One plate failed because of the fracture of the two pins located near the fracture line. One mesh broke vertically without pin separation, while all the pins of another mesh were fractured.

## 4 Discussion

This study sought to determine the differences in the initial fixation characteristics of four different treatment methods of mandibular fracture repair in cats and other relevant clinical parameters. Mechanical testing results demonstrated no differences in mechanical integrity between any of the treatment constructs. As expected, intact mandibles (control group) were stronger and stiffer than all treatment groups in this study, which has been previously reported, even when compared to titanium miniplates (14, 15). The application of absorbable implants was faster to perform than for the intraoral splints, and there was a higher incidence of root injuries in the mesh group (M) than in the plate group (P).

Bending moment, energy to failure, and maximum force were related to the strength of the construct. Bending moment is a measure of the force applied to the construct that causes angular deformation (28). All bending moments were significantly inferior to the bending moment of the intact bone. Based on the results of the present study, the modifications in the intraoral splints or the different number of pins in the mesh vs. plate group do not seem to substantially influence the mechanical characteristics. *In vitro* forces applied only to the canine tooth result in greater force at the fracture site than the resultant in clinical patients. During mastication the force is distributed over multiple teeth and applied closer to the fracture site (premolar and molar teeth) (28). Application of the force closer to the fracture site reduces the moment arm and, with the reduction of applied force due to avoidance of mastication, reduces the bending moment at the fracture site.

The mean maximum force of the four treatment constructs (78.31–99.88 N) is consistent with the estimated mean maximum bite force in cats at the level of the canine (73.3 N) and carnassial teeth (118.1 N) (27). The maximum force is like the one sustained by two different types of intraoral splints commonly used in dogs (crossover = 80.5 N; Stout = 51.8 N) (10). Although the exact masticatory force in awake cats has not been reported, it is unlikely that the masticatory forces reach the estimated maximum value immediately after surgery (14). The masticatory forces after repair of a mandibular fracture are reduced in humans and increase progressively during the following weeks after repair (29, 30). Furthermore, cats with maxillofacial trauma will have dietary (soft diet, feeding tube placement) and environmental modifications (avoidance of biting behaviors and rough play) for a minimum of 6 to 8 weeks to decrease load and mandibular motion during the initial stages of bone healing.

Mechanically, bending stiffness and deflection angle provide information regarding interfragmentary motion. Stiffness is relevant clinically because it measures how rigid the construct is and is a measurement of the fracture stability. Primary bone healing occurs with stiffer constructs, while secondary bone healing with callus formation is characteristic of more flexible (less stiff) constructs (28, 31). Interfragmentary micromotion in long bones stimulates greater bone healing than complete stability achieved with titanium plates (32). However, the stiffness of a construct or the interfragmentary motion that would be ideal to achieve healing in feline mandibles is unknown. In the present model, the stiffness of the four tested treatment groups was comparable and lower than that of intact mandibles. Intraoral splints are best indicated for transverse or oblique fractures that can be anatomically reduced (9, 11). In the present model, the fracture was not simulated by cracking the bone but by cutting the bone with a disk, which may have caused some bone loss and a bone gap in all treatment constructs, potentially reducing stability. Therefore, the results of the present study underestimate the mechanical characteristics of all treatment constructs.

The stiffness of intraoral splints in dogs increased with the incorporation of a greater dental surface in the splint (31). The crown of the teeth in cats is small, limiting the dental surface available for attachment of the bis-acryl composite. The standard technique (29, 31) to apply the intraoral splints in dogs was modified in the present study because of the different gingival and dental anatomy between dogs and cats. The wires were placed supragingivally and could not be tightly looped around the neck of the tooth secondary to the small or absent interdental spaces and the limited amount of gingiva typically found in cats. The height and width of the splint were homogeneous. The height was approximately the same as the height of the premolar and molar teeth. The thickness of the material applied affects its structural stiffness, but increasing the height and width may not be feasible if it interferes with mouth closing (limited by the height of the teeth and occlusion) and the function of the tongue. The inclusion of two teeth in the splint rostral and caudal to the fracture has previously been recommended (11). In clinical cases, the opposite mandibular canine tooth may be included in splint fabrication, but additional teeth cannot be incorporated into the splint in the caudal fragment. Dental adhesive agents increase the macro- and micromechanical retention of composites (33); however, bis-acrylic composite (Protemp Plus, 3 M ESPE) does not require the use of a bonding agent according to the manufacturer. In addition, a cadaveric study comparing splints for maxillomandibular fixation in cats showed more tooth fractures in the canine teeth when adhesives were applied on full vs. partial crown surfaces (34).

Constructs with low stiffness are not ideal for bone healing. However, there are limitations to improving stiffness when intraoral splints are applied. Absorbable implants perform similarly to intraoral splints, and the use of 6 (mesh) instead of 4 pins (plate) did not seem to improve the stiffness of the absorbable implants. More woven bone than lamellar bone formation was found in healed osteotomies in mandibles of dogs stabilized with absorbable implants compared to titanium plates, showing that absorbable implants were able to achieve bone healing despite the interfragmentary motion observed in the first weeks of the postoperative period in that study (35).

Statistical difference between the deflection angles of each treatment construct was not found in the present study, and only the Risdon configuration revealed a significantly higher deflection angle than the intact mandibles. Therefore, although not significant, a Risdon configuration will be a less stable technique allowing for more motion than the Stout configuration and the absorbable implants. The lower deflection angles with the mesh implant could be explained because of the positioning of the pins in the compression (ventral) and tension sides (dorsal) of the mandible. Only five mandibles remained for data analysis in three treatment groups, which may have affected the results.

The results of the present study suggest that primary bone healing is unlikely to occur in mandibular fractures treated with intraoral splints and absorbable implants because they have low stiffness and allow some degree of interfragmentary motion. This is consistent with the authors’ clinical experience with intraoral splints, but further studies are necessary to evaluate healing.

There was no correlation between the number of pins overlapping with the teeth and the mandibular canal, and the bending moment or stiffness. The bone available to anchor the screws may be insufficient when the mandibular canal is perforated. Constructs with screws in the mandibular canal are less stiff than when the screws engage in cortical bone (15). The results of the present study suggest that ultrasound-aided absorbable implants may be beneficial in areas with thin bone.

The mode of failure of the intraoral splints is consistent with previous studies (10, 31). However, the present study found that 25% of intraoral splints failed due to adhesive failure. The difficulty in looping the wire tightly around the neck and adapting it to the surface of teeth may have caused wire instability, thus affecting the bonding of the bis-acryl composite to the tooth. Furthermore, the dental surface available for attachment is limited as discussed above. This underscores the limitations and difficulty of placing intraoral splints in cats.

A recent study showed that the weakest point of ultrasound-aided absorbable implants is the plate (36) which is consistent with the mesh that fractured in the present study. Instead, the main mode of failure in the absorbable implants was the breakage of the pins inside the pilot hole, which is more likely related to the amount of polymer that fills out the pilot hole and the bone surface in which the polymer can diffuse. In the present study, 1.6 mm diameter x 5-mm-long pins were chosen arbitrarily. However, using 2.1 mm diameter and longer pins (up to 12 mm pins are available) might have resulted in a better and stronger construct, as the pin length and size affect the strength of the bond with bone (37). The drill used to create the pilot hole could injure the contents of the mandibular canal in the same way that titanium plates and screws can (14, 15). Neurological complications (paresthesia and neuropathic pain) secondary to screw placement in the mandibular canal have not been reported in animals but have been described in humans (38).

All surgical procedures were performed by the same investigator who had more experience in intraoral splinting than absorbable implant placement. The procedures were performed without an assistant. The placement of the absorbable implants was faster than the intraoral splints. This is consistent with the human medical literature, which showed that one of the advantages of ultrasound-activated absorbable implants is the reduction of surgical time for placement (25). The absorbable pins do not need any hole tapping, and the absorbable plates are easily adapted to the bone surface after softening in the warm bath. Additional reduction of the surgical time for the absorbable implants may be possible with experience and an assistant to help reduce the fracture fragments and perfect adaptation of the absorbable plate against the bone. Failure to keep contact between the plate or mesh and the bone prevents the insertion of the melting pins in the pilot holes. This may be the reason for the failure of one of the 4-hole absorbable plates during the cycling loading testing. The reduced time of the procedure would only be beneficial if the same healing were to occur with all the implants without further injury to important structures.

Dental trauma caused by implants is an important consideration when using internal fixation (17). Injury of the tooth pulp is associated with infection, inflammation, and failure of the repair (17). The pins in the present study were not long enough to obtain bicortical anchorage of the absorbable plate or mesh. However, the pilot holes likely reached deep enough to affect tooth roots. The higher incidence of root damage in the mesh group than the plate group was expected because two of the pins were placed in the dorsal half of the mandible and not in the ventral border of the mandible, the latter of which is considered to be the ideal location for plates (14). Plate contouring was helpful in avoiding trauma to the root of the canine tooth in some instances, but it was extremely challenging or impossible in many cases. Changing the direction of the pins (oblique rather than perpendicular placement) may avoid tooth roots in some instances. Previous studies showed that periodontal tissues and roots damaged by titanium screws often heal, provided there is no infection/inflammation and the injury only affects the cementum or dentin (39, 40). Histological evaluation of the healing and inflammation induced by absorbable pins into the bone of goats and sheep have shown minimal or absent inflammatory reaction (22, 41, 42). It is not known whether teeth suffering minimal (cementum or dentin) injury would heal the same way that they would if titanium screws were used. Thus, absorbable implants may at this moment not be the preferred option when the pilot holes cannot avoid tooth roots.

The mechanical stress that a construct sustains during bone healing depends on many factors such as masticatory forces, soft tissue attachment, type of fracture, influence of the other mandible, and increased stabilization by the callus formation in weeks following mandibular fracture repair (14, 36). The present mechanical model only considered forces sustained by the fractured fragments and the construct under cyclic loading and loading until failure. The repetitions during the cyclic loading are fewer than the ones occurring in an patient due to normal jaw movement. It did not consider the shearing load on the opposite mandible, and the bone loss caused by cutting with the diamond disk created a gap, which prevented load distribution between the bone fragments and any of the constructs. Mechanical models are useful to compare different constructs, but they do not account for the patient’s biological response. The data from mechanical studies only correspond to the immediate postoperative period (43). The forces applied in the present study and the cantilever bending model may not represent the exact forces that an intact or fractured mandible sustains during mastication or after surgical repair; therefore, direct extrapolation to clinical patients is not warranted. In addition, the freezing process may have had an impact on the mechanical properties of the materials.

The four treatment constructs tested in the present study are weaker and less stiff than intact mandibles. From a mechanical standpoint, the findings of this study suggest that absorbable implants may be sufficient to provide initial stabilization of mandibular body fractures in cats. In addition, the absorbable implants are faster to apply. However, tooth root injuries and perforation of the mandibular canal are likely to occur during the placement of the absorbable implants in the mandibular body. For these reasons, in the presence of teeth, Stout multiple loop interdental wiring with bis-acryl composite splinting would be the authors’ choice of treatment as it has a lower deflection angle (although not statistically significant) and less interfragmentary motion. Absorbable implants with larger and longer pins may be an option if there are missing teeth, as long as tooth roots and the mandibular canal can be avoided. In vivo studies are warranted to evaluate bone healing and the effect of stress during the initial weeks of healing.

## Data Availability

The raw data supporting the conclusions of this article will be made available by the authors, without undue reservation.

## References

[1] Knight R Meeson RL. Feline head trauma: a CT analysis of skull fractures and their management in 75 cats. J Feline Med Surg. (2019) 21:1120–6. doi: 10.1177/1098612X18819183, PMID: 30571454 PMC10814275

[2] Tundo I Southerden P Perry A Haydock RM. Location and distribution of craniomaxillofacial fractures in 45 cats presented for the treatment of head trauma. J Feline Med Surg. (2019) 21:322–8. doi: 10.1177/1098612X18776149, PMID: 29792378 PMC10814633

[3] Fisher CJ Cavanagh AA Liss D Adams T Marvel SJ Hall KE. Surgical interventions and outcome in a population of feline trauma patients. J Vet Emerg Crit Care. (2023) 33:337–47. doi: 10.1111/vec.13291, PMID: 37120709 PMC10350302

[4] Freeman A Southerden P. Mandibular fracture repair techniques in cats: a dentist's perspective. J Feline Med Surg. (2023) 25:1098612X231152521. doi: 10.1177/1098612X231152521, PMID: 36744847 PMC10812066

[5] Nicholson I Wyatt J Radke H Langley-Hobbs SJ. Treatment of caudal mandibular fracture and temporomandibular joint fracture-luxation using a bi-gnathic encircling and retaining device. Vet Comp Orthop Traumatol. (2010) 23:102–8. doi: 10.3415/VCOT-09-03-0034, PMID: 20151077

[6] Boudrieau RJ Kudisch M. Miniplate fixation for repair of mandibular and maxillary fractures in 15 dogs and 3 cats. Vet Surg. (1996) 25:277–91. doi: 10.1111/j.1532-950x.1996.tb01414.x, PMID: 8810018

[7] Kling K Marretta SM. Approach and Interfragmentary stabilization of caudal mandibular fracture in the cat. J Vet Dent. (2023) 40:81–8. doi: 10.1177/08987564221128663, PMID: 36177536

[8] Cetinkaya MA Yardimci C Kaya U. Lingual arch bar application for treatment of rostral mandibular body fractures in cats. Vet Surg. (2011) 40:457–63. doi: 10.1111/j.1532-950X.2011.00809.x, PMID: 21395619

[9] Kleftouri S Panagopoulou E Kouki MI. Fractures of the mandible in cats. Retrospective study of 23 cases. Hellenic J Companion Anim Med. (2017) 6:21.

[10] Kitshoff AM de Rooster H Ferreira SM Burger D Steenkamp G. The comparative biomechanics of the reinforced interdental crossover and the stout loop composite splints for mandibular fracture repair in dogs. Vet Comp Orthop Traumatol. (2013) 26:461–8. doi: 10.3415/VCOT-12-11-0140, PMID: 24081455

[11] Snyder CJ. Maxillofacial fracture repair using noninvasive techniques In: Verstraete FJM Lommer MJ Arzi B, editors. Oral and maxillofacial surgery in dogs and cats. 2nd ed. St Louis, MI: Elsevier (2020). 297–308.

[12] Guzu M Hennet PR. Mandibular body fracture repair with wire-reinforced interdental composite splint in small dogs. Vet Surg. (2017) 46:1068–77. doi: 10.1111/vsu.12691, PMID: 28759118

[13] Silva AM Souza WM Koivisto MB Barnabé Pde A Souza NT. Miniplate fixation for the repair of segmental mandibular defects filled with autogenous bone in cats. Acta Cir Bras. (2011) 26:174–80. doi: 10.1590/s0102-86502011000300004, PMID: 21537518

[14] Greiner CL Verstraete FJM Stover SM Garcia TC Leale D Arzi B. Biomechanical evaluation of two plating configurations for fixation of a simple transverse caudal mandibular fracture model in cats. Am J Vet Res. (2017) 78:702–11. doi: 10.2460/ajvr.78.6.702, PMID: 28541156

[15] Kot CCS Verstraete FJM Garcia TC Stover SM Arzi B. Biomechanical evaluation of locking versus nonlocking 2.0-mm malleable L-miniplate fixation of simulated caudal mandibular fractures in cats. Am J Vet Res. (2022) 83:43. doi: 10.2460/ajvr.22.03.0043, PMID: 35895785

[16] Bilgili H Kurum B. Treatment of fractures of the mandible and maxilla by mini titanium plate fixation systems in dogs and cats. Aust Vet J. (2003) 81:671–3. doi: 10.1111/j.1751-0813.2003.tb12533.x, PMID: 15086106

[17] Verstraete FJM Ligthelm AJ. Dental trauma caused by screws in internal fixation of mandibular osteotomies in the dog. Vet Comp Orthop Traumatol. (1992) 1992:104–8.

[18] El-Saadany WH Sadakah AA Hussein MM Saad KA. Evaluation of using ultrasound welding process of biodegradable plates for fixation of pediatric mandibular fractures. Tanta Dental J. (2015) 12:S22–9. doi: 10.1016/j.tdj.2015.08.003

[19] Noureldin MG Khalil AF Kotb AR Fahmy MH Abo Eleneen SED. Evaluation of resorbable mesh and plate with ultrasonic welded pins in the management of mandibular fracture in children (a comparative clinical study). Alex Dent J. (2016) 41:261–8. doi: 10.21608/adjalexu.2016.58038

[20] Lopez J Siegel N Reategui A Faateh M Manson PN Redett RJ. Absorbable fixation devices for pediatric Craniomaxillofacial trauma: a systematic review of the literature. Plast Reconstr Surg. (2019) 144:685–92. doi: 10.1097/PRS.0000000000005932, PMID: 31461027

[21] Buijs GJ Van der Houwen EB Stegenga B. Mechanical strength and stiffness of the biodegradable SonicWeld Rx osteofixation system. J Oral Maxillofacial Surg. (2009) 67:782–7. doi: 10.1016/j.joms.2008.07.022, PMID: 19304035

[22] Mai R Lauer G Pilling E Jung R Leonhardt H Proff P. Bone welding--a histological evaluation in the jaw. Ann Anat. (2007) 189:350–5. doi: 10.1016/j.aanat.2007.02.023 17695991

[23] Pilling E Maia R Theissig F Stadlinger B Loukota R Eckelt U. An experimental in vivo analysis of the resorption to ultrasound activated pins (sonic weld) and standard biodegradable screws (resorb X) in sheep. Br J Oral Maxillofac Surg. (2007) 45:447–50. doi: 10.1016/j.bjoms.2006.12.002, PMID: 17218041

[24] Gareb B van Bakelen NB Driessen L Buma P Kuipers J Grijpma DW. Biocompatibility and degradation comparisons of four biodegradable copolymeric osteosynthesis systems used in maxillofacial surgery: a goat model with four years follow-up. Bioact Mater. (2022) 17:439–56. doi: 10.1016/j.bioactmat.2022.01.015, PMID: 35386449 PMC8961280

[25] Lee JH Park JH. The clinical usefulness of ultrasound-aided fixation using an absorbable plate system in patients with zygomatico-maxillary fracture. Arch Plast Surg. (2013) 40:330–4. doi: 10.5999/aps.2013.40.4.330, PMID: 23898427 PMC3723991

[26] Reiter AM Gracis M. Management of dental and oral trauma In: Reiter AM Gracis M, editors. Manual of canine and feline dentistry and oral surgery. 4th ed. Gloucester: BSAVA (2018). 196–244.

[27] Kim SE Arzi B Garcia TC Verstraete FJM. Bite forces and their measurement in dogs and cats. Front Vet Sci. (2018) 5:76. doi: 10.3389/fvets.2018.00076, PMID: 29755988 PMC5932386

[28] Geddes AT Thatcher GP Hetzel S McCabe RP Vandereby R Jr Snyder CJ. Biomechanical testing of a calcium phosphate-phosphoserine-based mineral-organic adhesive for non-invasive fracture repair of mandibular fractures in dogs. Front Vet Sci. (2020) 7:59. doi: 10.3389/fvets.2020.00059, PMID: 32181262 PMC7058112

[29] Kryeziu K Prekazi-Loxha M Hajdari B Salihu L Vela-Gaxha Z Stubljar D. Masticatory muscles activity in patients with mandibular angle fractures: a literature review on which procedure to use to reverse the best masticatory muscles functionality. Heliyon. (2023) 9:e15024. doi: 10.1016/j.heliyon.2023.e15024, PMID: 37096003 PMC10121927

[30] Gheibollahi H Aliabadi E Khaghaninejad MS Mousavi S Babaei A. Evaluation of bite force recovery in patients with maxillofacial fracture. J Craniomaxillofac Surg. (2021) 5182:00074–3. doi: 10.1016/j.jcms.2021.02.017 33653602

[31] Lothamer C Snyder CJ Duenwald-Kuehl S Kloke J McCabe RP Vanderby R Jr. Crown preservation of the mandibular first molar tooth impacts the strength and stiffness of three non-invasive jaw fracture repair constructs in dogs. Front Vet Sci. (2015) 2:18. doi: 10.3389/fvets.2015.00018, PMID: 26664947 PMC4672188

[32] Glatt V O'Toole R Mehta S Ricci W Nauth A Schemitsch E. Great debates in trauma biomechanics. OTA Int. (2023) 6:e249. doi: 10.1097/OI9.0000000000000249, PMID: 37168029 PMC10166369

[33] Domnick ED. Use of composite restoration materials. J Vet Dent. (2014) 31:280–8. doi: 10.1177/089875641403100413, PMID: 30870961

[34] Hoffer M Manfra Marretta S Kurath P Johnson A Griffon D Schaeffer D. Evaluation of composite resin materials for maxillomandibular fixation in cats for treatment of jaw fractures and temporomandibular joint luxations. Vet Surg. (2011) 40:357–68. doi: 10.1111/j.1532-950X.2010.00782.x, PMID: 21244442

[35] Sverzut CE Kato RB Rosa AL Trivellato AE Sverzut AT da Silveira KM. Comparative study of bone repair in mandibular body osteotomy between metallic and absorbable 2.0 mm internal fixation systems. Histological and histometric analysis in dogs: a pilot study. Int J Oral Maxillofac Surg. (2012) 41:1361–8. doi: 10.1016/j.ijom.2012.04.012, PMID: 22633468

[36] Gareb B Roossien CC van Bakelen NB Verkerke GJ Vissink A Bos RRM. Comparison of the mechanical properties of biodegradable and titanium osteosynthesis systems used in oral and maxillofacial surgery. Sci Rep. (2020) 10:18143. doi: 10.1038/s41598-020-75299-9, PMID: 33097757 PMC7584639

[37] Lopez A Ayyachi T Brouwers T Åberg J Wistrand AF Engqvist H. 1-year pullout strength and degradation of ultrasound welded vs tapped craniomaxillofacial fixation screws. Polym Test. (2022) 109:107519. doi: 10.1016/j.polymertesting.2022.107519

[38] Phillips C Essick G. Inferior alveolar nerve injury following orthognathic surgery: a review of assessment issues. J Oral Rehabil. (2011) 38:547–54. doi: 10.1111/j.1365-2842.2010.02176.x, PMID: 21058973 PMC3094736

[39] Brisceno CE Rossouw PE Carrillo R Spears R Buschang PH. Healing of the roots and surrounding structures after intentional damage with miniscrew implants. Am J Orthod Dentofacial Orthop. (2009) 135:292–301. doi: 10.1016/j.ajodo.2008.06.023, PMID: 19268826

[40] Lv Y Zhang Z Su Y Yuan P Ma W Huang W. Healing of root and surrounding periodontium after root damage with miniscrew implants: a histomorphologic study in dogs. Clin Oral Investig. (2018) 22:1103–11. doi: 10.1007/s00784-017-2194-z, PMID: 28861710

[41] Heidenreich D Langhoff JD Nuss K Kluge K Kämpf K Zlinsky K. The use of BoneWelding® technology in spinal surgery: an experimental study in sheep. Eur Spine J. (2011) 20:1821–36. doi: 10.1007/s00586-011-1799-1, PMID: 21523457 PMC3207336

[42] Schneider M Seinige C Pilling E Rasse M Loukota R Stadlinger B. Ultrasound-aided resorbable osteosynthesis of fractures of the mandibular condylar base: an experimental study in sheep. Br J Oral Maxillofac Surg. (2012) 50:528–32. doi: 10.1016/j.bjoms.2011.10.003, PMID: 22078939

[43] Augat P Hast MW Schemitsch G Heyland M Trepczynski A Borgiani E. Biomechanical models: key considerations in study design. OTA Int. (2021) 4:e099. doi: 10.1097/OI9.0000000000000099, PMID: 37608858 PMC10441683

